# Supplementary material for: High throughput 3D gel-based neural organotypic model for cellular assays using fluorescence biosensors
Source: Commun Biol. 2022 Nov 12;5:1236. doi: 10.1038/s42003-022-04177-z (PMC9653447; doi:10.1038/s42003-022-04177-z)
Supplement: Supplementary file 2 — Supplementary Information [file 42003_2022_4177_MOESM2_ESM.pdf]

High throughput 3D gel-based neural organotypic model for cellular assays using fluorescence biosensors

Srikanya Kundu[1], Molly E Boutin[1], Caroline E Strong[1], Ty Voss[1] and Marc Ferrer[1]*

[1] 3D Tissue Bioprinting Laboratory, National Center for Advancing Translational Sciences, National Institute of Health, 9800 Medical Center Dr, Rockville, MD 20850, USA

Supplementary Figures

Supplementary Figure 1: Cell titer Glow Viability assay for 3D gel-based iDopas/iAstros coculture model.

a)

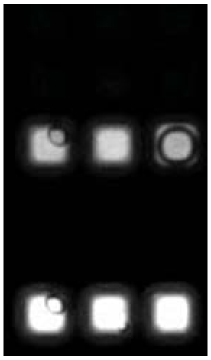

b)

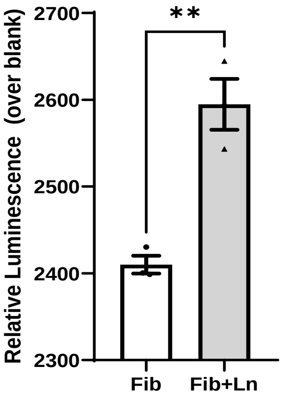

CellTiterGlo viability assay for 3D gel-based iDopas/iAstros coculture model. The viability of the cells in 3D coculture model was confirmed via CellTiterGlo 3D assay. a) Viable cells are shown by the brightness of the luminescent image from the 384 well plate (n=3). Without cells, the gel-matrix showed no luminescence (first and third rows from top) either with or without laminin. The bright luminescent from the second row indicated the presence of live cells in the wells (second row from top). The addition of laminin increased the brightness from each well indicating higher number of viable cells in each well (last row). b) Quantitation of the relative luminescent against the blank confirmed that the laminin promoted cell viability more than doubled in our 3D fibrin gel-based model.

Supplementary Figure 2: Optimization of MOI (multiplicity of infection) for Adeno Associated Virus serotype on human iPSC derived dopaminergic neuronal co-culture.

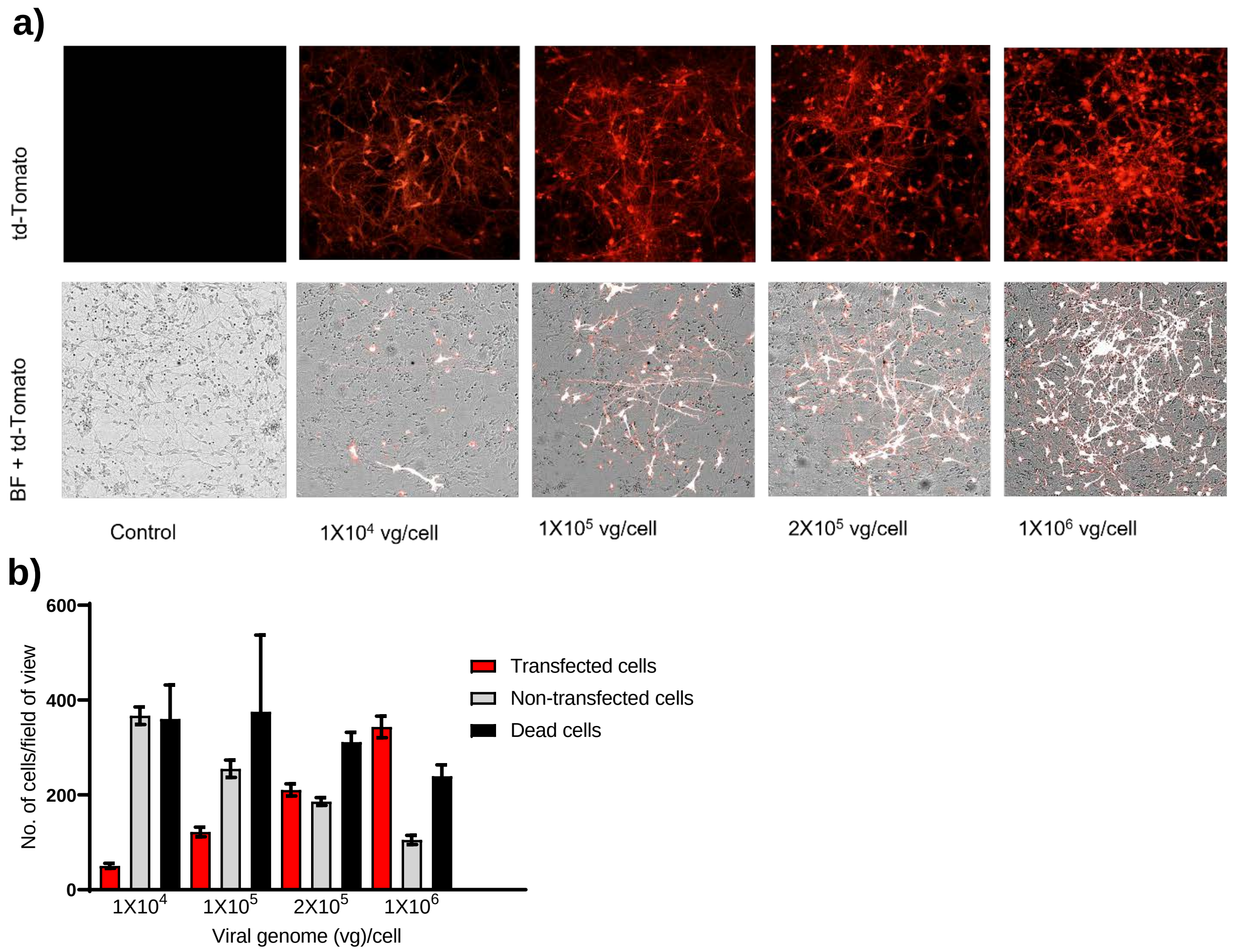

We calibrated the MOI of the Adeno-associated virus serotype 9 on our iPSC derived human dopaminergic neuronal coculture in 2D format. We transfected our 2D coculture with AAV9 tagged with td-Tomato with serial dilution of number of viral genomes (vg): i) Control with no virus; ii) 1×104 vg/cells; iii) 1×105 vg/cells; iv) 2×105 vg/cells; iv) 1×106 vg/cells. The representative images of the confocal red-florescent (560/600 filter) (top row) and the merged with brightfield (bottom row) from each dilution were shown in figure a). b) The quantitative analysis was presented as a bar plots from each group (n=3 wells). The number of red cells were counted as virus transfected cells, the non-transfected cells were spotted by their dark gray color on brightfield images with the presence of their neurites and the black dots spots without any extension on the brightfield images were considered as dead cells. The average of multiple view from single well and 3 wells from each dilution group were plotted with their s.e.m. (number of replicates, n=3). The highest number of transfected cells against the lowest number of non-transfected cells ratio with moderate number of dead cells group was chosen as multiplicity of infection per cells for

iPSC derived neuronal coculture. The chosen 1×106 vg/cell MOI was used through out of our experiments for both dopaminergic and glutamatergic 2D and 3D cocultures.

Supplementary Figure 3: Validation of AAV9-CAG-GCamP6f transfected against Calbryte calcium dye-based calcium fluorescence measurements of iDopas/iAstros 2D coculture using a FLIPR screening instrument.

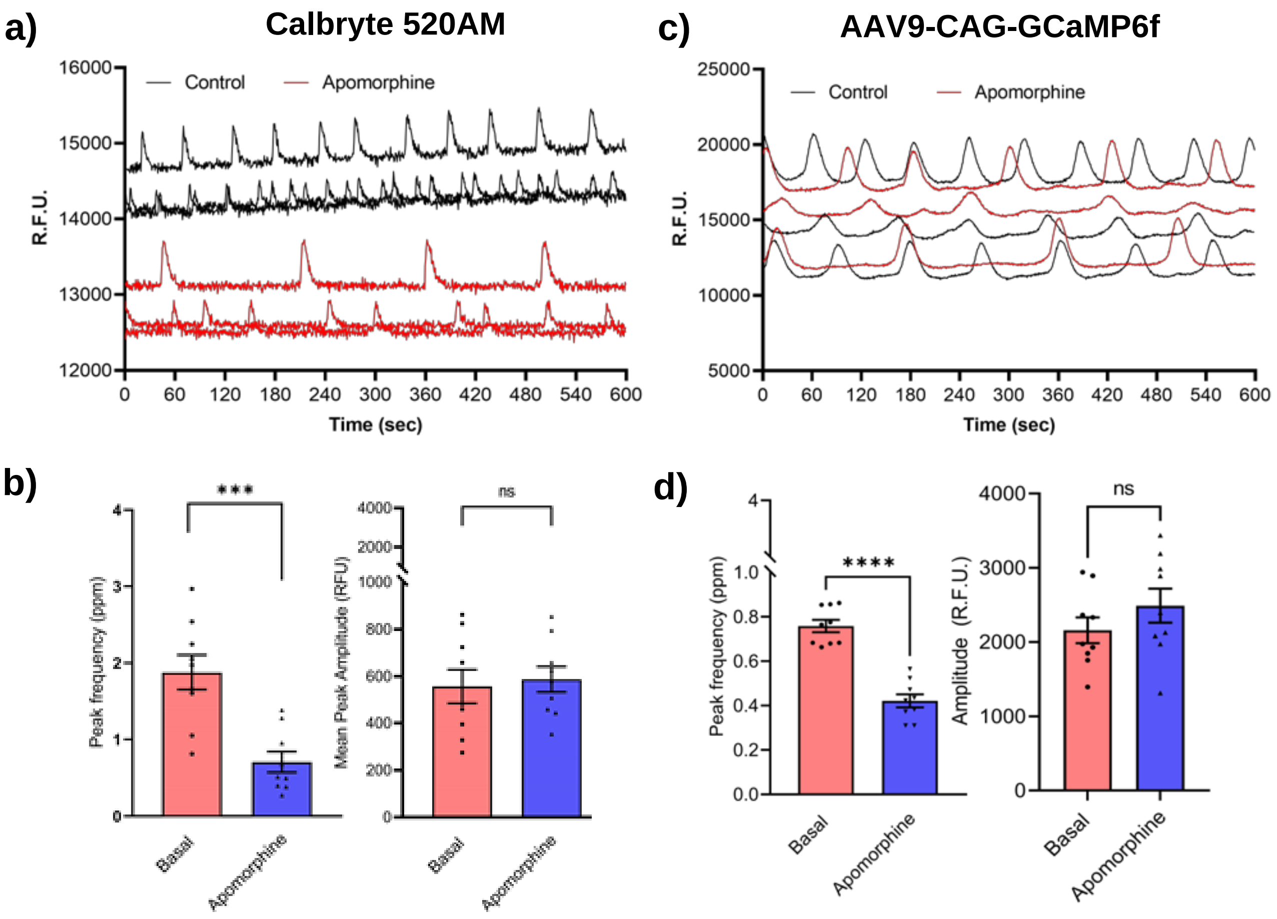

Calbryte 520AM, using a well-based FLIPR reader. Representative raw fluorescence unit (R.F.U.) traces of spontaneous calcium waves (black traces) and their corresponding pharmacological modulations with D2-receptor agonist, apomorphine (red traces) using calbryte 520AM (a) and GCaMP6f biosensors (b) respectively. The calcium dynamics were measured for 600sec with 0.6sec acquisition interval in FLIPR from n=3 wells/group. b) & c) The calcium peaks properties including peak frequency per sec and mean peak amplitude were quantitated before and after the drug (apomorphine) applications using the FLIPR's analysis software Screenwork 5.1. GCaMP6f biosensors produced slower

spontaneous calcium dynamics over time in terms of peak frequency than the calbryte 520AM. Both showed negative modulation by apomorphine application after 30 minutes, with no significant changes with peak amplitude. Error bar s.e.m. , n= 9

Supplementary Figure 4: Verification of ChrimsonR-opsin expression by td-Tomato fluorescence in representative images of 2D and 3D neuronal co-culture

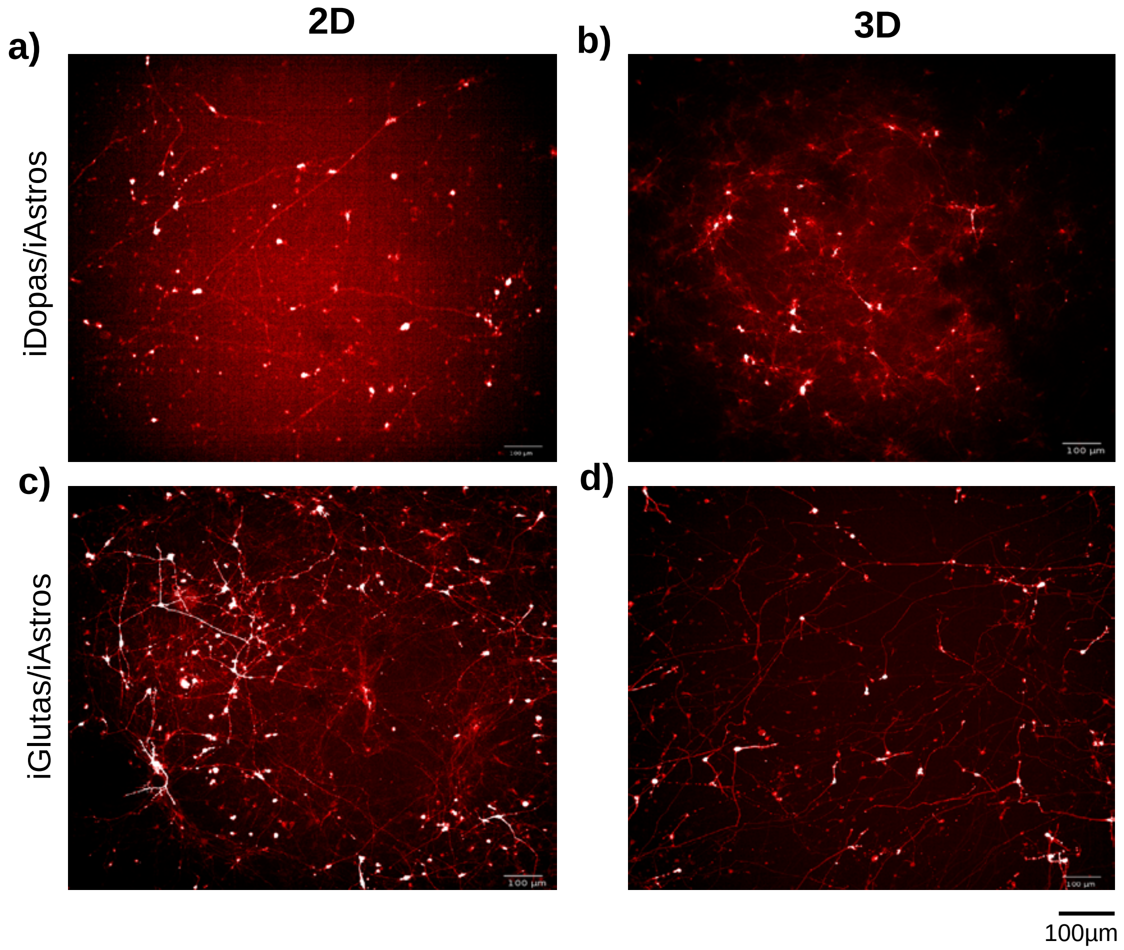

Verification of ChrimsonR expression via td-Tomato fluorescence in representative images from 2D and 3D neuronal co-culture. The imaging planes for measuring calcium dynamics over time were selected by checking the expression label of ChrimsonR via td-Tomato fluorescence intensity. A satisfactory expression of ChrimsonR on a single plane of view ensured the sufficient evoked activity in the neuronal network both in 2D (a) and 3D (b) system.

Supplementary Figure 5: Presence of μ-opioid receptors on human iPSC derived dopaminergic and Glutamatergic neuronal coculture.

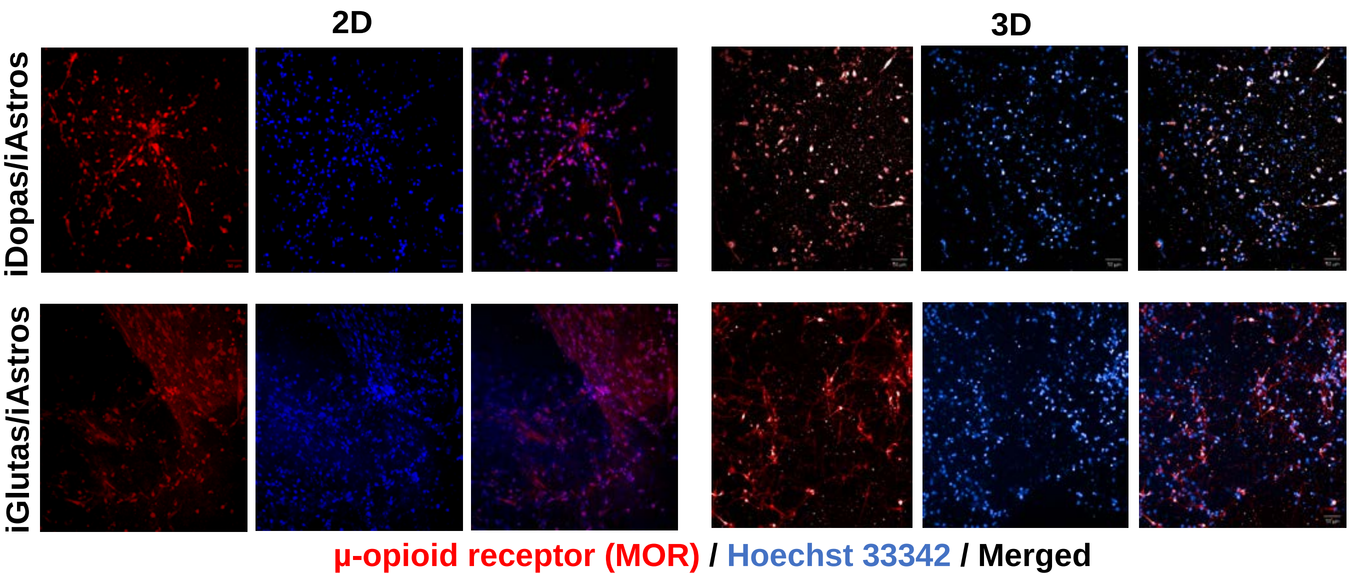

Presence of μ-opioid receptors on human iPSC derived dopaminergic and Glutamatergic neuronal coculture. Immunohistochemistry analysis of neural co-cultures to assess expression of μ-opioid receptors. PFA fixed cultures were stained with anti-mu-opioid receptor antibody (red) and Hoechst 33342 nuclear staining (blue), showing presence of μ-opioid receptors in the cell bodies and neurites of both the neurons type co-cultures, in both 2D and 3D models. Images are from single plane confocal fluorescence acquisition for both 2D and 3D co-cultures. Scale bar 50μm.mag 10X

Supplementary Figure 6: Validation of non-lethality of pharmacological treatment (apomorphine) after 24 hours of drug washout with iDopas/iAstros co-culture in 2D and 3D gel-based system

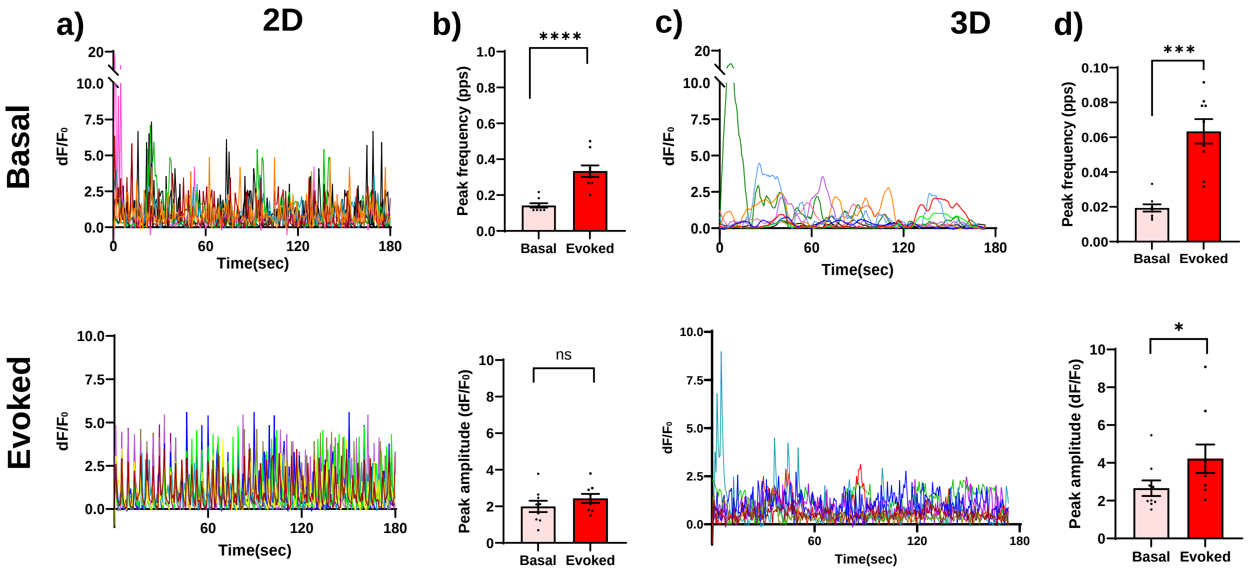

Randomly picked wells from apomorphine treatment group after 24 hrs of drug washout were presented as example of functional validation of non-lethality of used drug dose. (a & c) The example traces of single cell calcium activity showed the normal calcium dynamic of healthy iDopas/iAstros co-culture in 2D and 3D format respectively. (b & d) Quantification of calcium peak frequency per sec and peak amplitude confirmed that both the culture reached their respective pre-treatment calcium dynamics. And the system was capable of re-stimulation and significantly elevated evoked activity were also matches with the respective pre-treatment label. Scale bar:60sec, * = $p<0.5$; *** = $p<0.001$; **** = $p<0.0001$, n.s.= not significant.
