# Supplementary material for: High throughput 3D gel-based neural organotypic model for cellular assays using fluorescence biosensors
Source: Commun Biol. 2022 Nov 12;5:1236. doi: 10.1038/s42003-022-04177-z (PMC9653447; doi:10.1038/s42003-022-04177-z)
Supplement: Supplementary file 3 — Description of Additional Supplementary Files [file 42003_2022_4177_MOESM3_ESM.pdf]

Description of Additional Supplementary Files

File name: Supplementary Data 1

Description: The source data behind the graphs in the paper (main figures)

File name: Supplementary Data 2

Description: The source data behind the graphs in the paper (supplementary figures)

Supplementary Movies

Supplementary movie 1: 3D rendering of x-z cross-section of gel-based neuronal model.

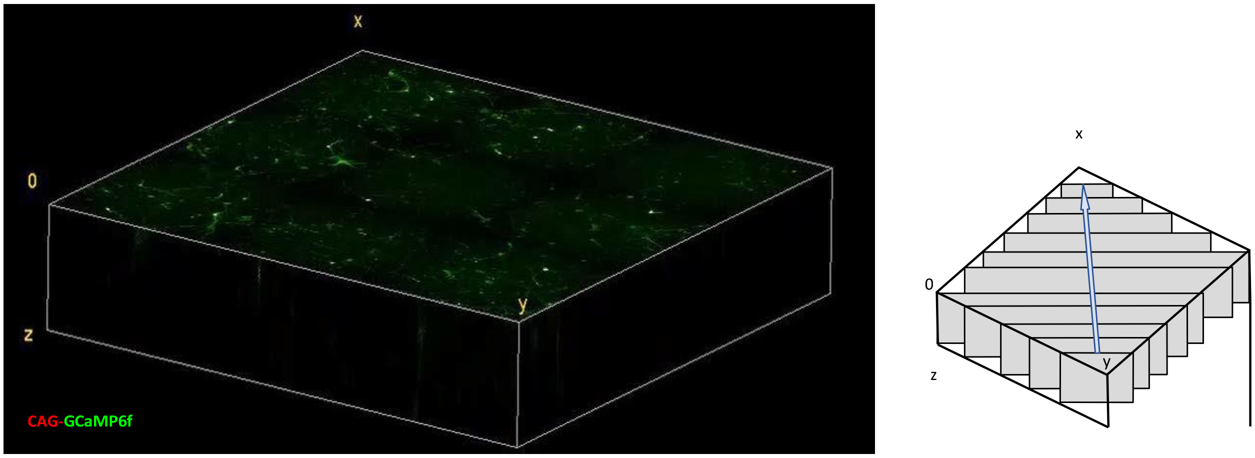

3D rendering movie of x-z cross-sections sequences of genetically encoded calcium biosensors, CAG-GCaMP6f (green fluorescence) activity across the y plane from 3D fibrin gel iDopas/iAstros neuronal coculture model

Supplementary movie 2: Spatial assessments of ‘in-network’ neuronal dynamics of 3D gel-based neuronal model.

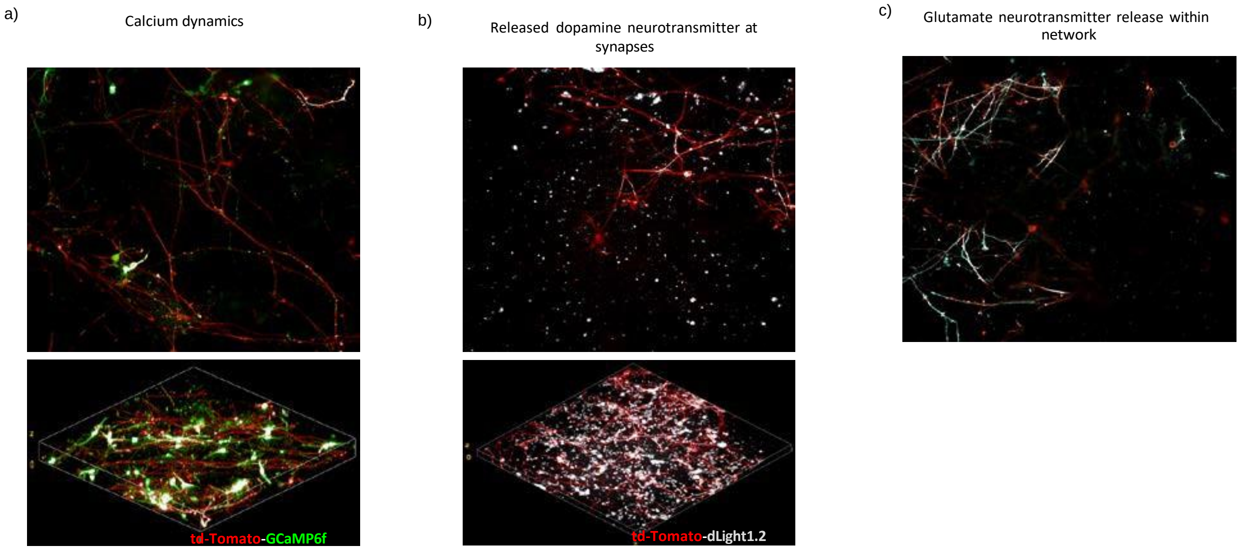

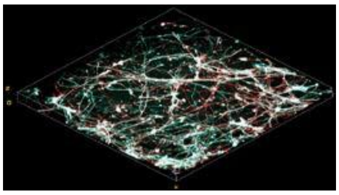

The images were taken with 10X air objective under confocal microscope from 3D fibrin gel neuronal models. a)Top row: z-stack images of calcium activity via CAG-GCaMP6f (green) merged with the expression of ChrimsonR via td-Tomato (red). Bottom row: 3D rendering and reconstruction of above z-stack images. b)Top row: z-stack images of released dopamine neurotransmitter via hSyn-dLight1.2 biosensor (white) at the synapses merged with merged with the expression of ChrimsonR via td-Tomato (red). Bottom row: 3D rendering and reconstruction of above z-stack images. c)Top row: z-stack images of 'in-network' released glutamate neurotransmitter via hSyn-iGluSnFr (sea green) along the neurites merged with the expression of ChrimsonR via td-Tomato (red). Bottom row: 3D rendering and reconstruction of above z-stack images. 10X
