# Supplementary material for: High throughput 3D gel-based neural organotypic model for cellular assays using fluorescence biosensors
Source: Commun Biol. 2022 Nov 12;5:1236. doi: 10.1038/s42003-022-04177-z (PMC9653447; doi:10.1038/s42003-022-04177-z)
Supplement: Supplementary file 7 — Reporting Summary [file 42003_2022_4177_MOESM7_ESM.pdf]

nature portfolio

Corresponding author(s): Marc Ferrer

Last updated by author(s): October 11, 2022

Reporting Summary

Nature Portfolio wishes to improve the reproducibility of the work that we publish. This form provides structure for consistency and transparency in reporting. For further information on Nature Portfolio policies, see our Editorial Policies and the Editorial Policy Checklist.

Statistics

For all statistical analyses, confirm that the following items are present in the figure legend, table legend, main text, or Methods section.

n/a	Confirmed	
☐	☒	The exact sample size (*n*) for each experimental group/condition, given as a discrete number and unit of measurement
☐	☒	A statement on whether measurements were taken from distinct samples or whether the same sample was measured repeatedly
☐	☒	The statistical test(s) used AND whether they are one- or two-sided<br>*Only common tests should be described solely by name; describe more complex techniques in the Methods section.*
☐	☒	A description of all covariates tested
☐	☒	A description of any assumptions or corrections, such as tests of normality and adjustment for multiple comparisons
☐	☒	A full description of the statistical parameters including central tendency (e.g. means) or other basic estimates (e.g. regression coefficient) AND variation (e.g. standard deviation) or associated estimates of uncertainty (e.g. confidence intervals)
☐	☒	For null hypothesis testing, the test statistic (e.g. *F*, *t*, *r*) with confidence intervals, effect sizes, degrees of freedom and *P* value noted<br>*Give P values as exact values whenever suitable.*
☒	☐	For Bayesian analysis, information on the choice of priors and Markov chain Monte Carlo settings
☐	☒	For hierarchical and complex designs, identification of the appropriate level for tests and full reporting of outcomes
☒	☐	Estimates of effect sizes (e.g. Cohen's *d*, Pearson's *r*), indicating how they were calculated

Our web collection on statistics for biologists contains articles on many of the points above.

Software and code

Policy information about availability of computer code

Data collection	Data processing for single cellular calcium dynamics: LC-Pro plug-in
Data analysis	The acquisition plane was determined by the satisfactory expression of ChrimsonR via td-Tomato fluorescence. After the selection of imaging plane, a series of 300 time-lapsed images were captured over a period of 180 sec with 0.6sec interval by Harmony v5.1 software (PerkinElmer). The time-lapse images were stacked to provide maximum intensity projection and transferred to ImageJ image processing software. Then ROIs were automatically selected centering on the neuronal soma by the 'LC-Pro' plug-in [91] in ImageJ by providing the ROI diameter (pixel) 30, frame rate (fps) 1.6 and for intensity cutoff threshold with p-value 0.05. Finally, the raw intensity values of the calcium signal were extracted from selected ROIs. For the analysis, each ROIs data were then transferred to 'Origin-Pro 9.0'(OriginLab). Single neuronal dynamics were quantified via the calcium signal peak frequency and peak amplitude. The peaks were detected over time by 'positive maximum intensity peak finding method' using 'batch peak processing algorithm', with second derivatives of individual ROI's raw fluorescence intensities (r.f.u.) as a change in fluorescence intensity from initial (dF) [92]. Because of sparsity of event's appearance in our 3D system and to improve the single to noise ratio, we used second derivatives of r.f.u. After thresholding the base line, the estimation of initial fluorescence level (F0) for each ROIs was calculated by averaging the r.f.u. over full acquisition length and then the amplitude was normalized to dF/F0 for respective ROIs. We did not used initial image frame's mean fluorescent intensity as our F0, because in both of 2D and 3D model on every image frame we had GCaMP6f activated fluorescence intensity from cells to some extent. We never got completely 'no signal' in any frame. The oscillations of calcium waves (dF/F0) from 3-4 example traces from each experimental group were presented in figure 4b for 2D and figure 4e for 3D system. The peak frequencies (peak per sec) and mean peak amplitudes from each ROIs, minimum of 3 ROIs per well from 3 biologically replicated wells per plate and 3 technical replicated plates per group were plotted as bar graph. We repeated the same analysis for each drugs treated group.

For manuscripts utilizing custom algorithms or software that are central to the research but not yet described in published literature, software must be made available to editors and reviewers. We strongly encourage code deposition in a community repository (e.g. GitHub). See the Nature Portfolio guidelines for submitting code & software for further information.

Data

Policy information about availability of data

All manuscripts must include a data availability statement. This statement should provide the following information, where applicable:

- Accession codes, unique identifiers, or web links for publicly available datasets
- A description of any restrictions on data availability
- For clinical datasets or third party data, please ensure that the statement adheres to our policy

The dataset generated during and/or analyzed during the current study are available from the corresponding authors on reasonable request.

Human research participants

Policy information about studies involving human research participants and Sex and Gender in Research.

Reporting on sex and gender	N/A
Population characteristics	N/A
Recruitment	N/A
Ethics oversight	N/A

Note that full information on the approval of the study protocol must also be provided in the manuscript.

Field-specific reporting

Please select the one below that is the best fit for your research. If you are not sure, read the appropriate sections before making your selection.

☒ Life sciences ☐ Behavioural & social sciences ☐ Ecological, evolutionary & environmental sciences

For a reference copy of the document with all sections, see nature.com/documents/nr-reporting-summary-flat.pdf

Life sciences study design

All studies must disclose on these points even when the disclosure is negative.

Sample size	Each bar graphs were plotted in GraphPad Prism 9.0 as mean±s.e.m. across 4 experimental groups, i) Basal, ii) Basal after respective drug treatment, iii) Evoked, iv) Evoked after corresponding drug treatment. Each experiment was repeated 3 times as biological replicates, and for each biological replicate, 3 wells were included for each treatment condition, as technical replicates. For measurements of single cellular calcium dynamics, each dot in the bar plots represents the 'n' = number of ROIs or cells per well. 3-5 cells from each well, autonomously picked by the LC-Pro 'ROI measurement algorithm' with maximum change in intensities over the series of 300 time-lapsed images for a period of 180sec, were selected for measurements. For the experiments to measure neurotransmitter levels, each dot in the bar plots represents the 'n' = number of wells. Total of 9 wells were accounted-for (3 biological × 3 technical replicates) per experimental group. Mean fluorescence intensities per well were obtained by averaging the fluorescence intensity of 5 'field of view' of epifluorescence images in the 2D cultures and 5 'field of views' of confocal z-stacks (max-projection) images in the 3D models.
Data exclusions	N/A
Replication	Experiments throughout the paper were mostly conducted at different dates over the period of 2 years with neuronal iPSC derived cells from different lots. Lots number mentioned in the methods section.
Randomization	Random selection of ROIs/cells for single cell calcium dynamics measurement were performed by LC-Pro software with plug-in algorithmic selection based on the maximum change/fluctuation in GCaMP6f fluorescent intensities over the period of 180 data points.
Blinding	N/A

Reporting for specific materials, systems and methods

We require information from authors about some types of materials, experimental systems and methods used in many studies. Here, indicate whether each material, system or method listed is relevant to your study. If you are not sure if a list item applies to your research, read the appropriate section before selecting a response.

Materials & experimental systems

n/a	Involved in the study
☐	☒ Antibodies
☒	☐ Eukaryotic cell lines
☒	☐ Palaeontology and archaeology
☒	☐ Animals and other organisms
☒	☐ Clinical data
☒	☐ Dual use research of concern

Methods

n/a	Involved in the study
☒	☐ ChIP-seq
☒	☐ Flow cytometry
☒	☐ MRI-based neuroimaging

Antibodies

Antibodies used

1. Tyrosine Hydroxylase (TH) (abchem, ab76442, anti-chicken, 1:200 dilution)
2. Glia Fibrillary Acidic Protein (GFAP) (Dako, Z0334, anti-rabbit, 1:1000 dilution)
3. Microtubule Associated Protein 2 (MAP2) (Sigma, M4402, anti-mouse, 1:250 dilution)
4. Postsynaptic Density Protein 95 (PSD95) (Thermofisher, MA1-045, anti-mouse, 1:100 dilution)
5. Neuronal Nuclear Protein (NeuN) (EMD Millipore, MAB377, 1:200 dilution)
6. mu-opioid receptor antibody (MOR) (abchem, ab10275, anti-rabbit, 1:200 dilution)
7. Hoechst 33342 (ThermoFisher, 62249, 1:2000 dilution)
8. Goat host selective (anti-mouse IgG/anti-rabbit IgG/anti-chicken/anti-rat IgG), Highly Cross-Adsorbed Secondary Antibody with Alexa Fluor 488/ 567/647 (ThermoFishers, 1:500 dilution)
9. Goat anti-mouse IgG (H+L) Alexa Fluor 488, Highly Cross-Adsorbed Secondary Antibody (ThermoFishers, 1:500 dilution)

Validation

Antibodies were validated in-house prior to use by testing in different tissue types for specificity. Antibodies that showed reactivity in inappropriate receptor types were not used in the study.
